# Impact of thyroid cancer treatment on assisted reproductive technology outcomes in women with infertility

**DOI:** 10.1007/s10815-021-02204-2

**Published:** 2021-04-26

**Authors:** Ning Huang, Lin Zeng, Jie Yan, Hongbin Chi, Jie Qiao

**Affiliations:** 1 Department of Obstetrics and Gynecology, Center for Reproductive Medicine, Peking University Third Hospital, 49 North Garden Rd, Beijing, 100191 China; 2 Clinical Epidemiology Research Center, Peking University Third Hospital, 49 North Garden Rd, Beijing, 100191 China

**Keywords:** Thyroid cancer, Assisted reproductive technology, Women with infertility, Radioactive iodine treatment

## Abstract

**Purpose:**

We investigated the effect of different surgical procedures and radioactive iodine treatment (RAIT) on in vitro fertilization/intracytoplasmic sperm injection (IVF/ICSI) outcomes and evaluated whether possible risk factors, including age, thyroid-stimulating hormone (TSH) levels, and thyroid antibody positivity, were associated with adverse IVF/ICSI outcomes.

**Methods:**

This retrospective study included 76 women with infertility who had received thyroid cancer (TC) treatment among 137,698 infertile women who underwent IVF/ICSI cycles at the Peking University Third Hospital between 2010 and 2019. Clinical pregnancy and live birth rates were assessed.

**Results:**

We found that the clinical pregnancy and live birth rates in women who underwent partial thyroidectomy were 7- and 6-fold higher, respectively, than those in women who underwent total thyroidectomy. We observed no significant differences in the clinical pregnancy and live birth rates between the RAIT and non-RAIT groups, even after adjusting for age, TSH levels, surgical treatment, and thyroid antibody positivity. Multivariate logistic regression analysis showed that age and TSH levels were not associated with decreased clinical pregnancy and live birth rates. Women with thyroid antibody positivity had significantly lower clinical pregnancy and live birth rates than women without thyroid antibody positivity.

**Conclusion:**

Our study showed lower clinical pregnancy and live birth rates in women who underwent total thyroidectomy than in women who underwent partial thyroidectomy. Thyroid antibody positivity is an important risk factor for adverse IVF/ICSI outcomes in women who have received TC treatment.

## Introduction

Several studies have demonstrated a significant association between reproductive factors and the risk of thyroid cancer (TC), and a higher prevalence of TC has been observed in women with infertility than in those without infertility [1–3]. For women with TC complicated by infertility, a careful balance between TC and assisted reproductive treatment is required. However, few studies have evaluated the impact of different TC treatments on in vitro fertilization/intracytoplasmic sperm injection (IVF/ICSI) outcomes.

Sufficient thyroid hormone secretion and a normal thyroid gland regulatory capacity are essential for follicular growth, pregnancy maintenance, and fetal development [4]. Both hyperthyroidism and hypothyroidism during pregnancy may be associated with adverse pregnancy outcomes [5]. Partial or total thyroidectomy combined with thyroid hormone suppression remains the standard treatment for patients with TC. A total or partial reduction in thyroid function after thyroidectomy and prolonged thyroid hormone-suppression therapy to reduce tumor recurrence may affect reproductive function and increase the risk of adverse pregnancy outcomes. The degree of thyroid function deficiency and thyroid hormone replacement dose differ between patients following total and partial thyroidectomy. Whether different surgical procedures trigger different pregnancy outcomes in women with TC remains unknown.

Except for thyroid function, an increasing number of recent studies began to focus on the impact of thyroid autoimmunity on the IVF/ICSI outcomes. Thyroglobulin, serving as a template and thyroid peroxidase, functioning as a key enzyme in thyroid hormone biosynthesis, constitutes the major thyroid autoantigens involved in the pathophysiology of thyroid autoimmunity [6]. Thyroid antibodies, mainly thyroid peroxidase antibody (TPOAb) and thyroglobulin antibody (TGAb), are important serum markers for diagnosing Hashimoto’s thyroiditis. The association between thyroid antibodies and IVF/ICSI outcomes has been demonstrated in some studies; however, the results are still controversial [7]. There are a few studies analyzing the effect of thyroid antibodies on IVF/ICSI outcomes in patients with TC although several studies have reported an increased risk of TC in patients positive for thyroid antibodies [8–10].

Radioactive iodine treatment (RAIT) after thyroidectomy is frequently used as an adjuvant therapy for the ablation of thyroid remnants. Although the 2017 American Thyroid Association guidelines recommend that pregnancy should be deferred for 6 months after RAIT, the results of studies that explored the correlation between RAIT and pregnancy outcomes are still controversial [5, 11–13]. Studies have reported that RAIT affects ovarian function and is associated with oligomenorrhea and early-onset menopause [14, 15]. However, the effect of RAIT on follicular development and IVF/ICSI outcomes remains unknown.

This study aimed to investigate the effect of different types of surgical procedures and RAIT on IVF/ICSI outcomes in women with TC and analyze whether possible risk factors, including age, thyroid-stimulating hormone (TSH) levels, and thyroid antibody positivity, were associated with adverse IVF/ICSI outcomes.

## Materials and methods

### Study population

**Fig. 1:**
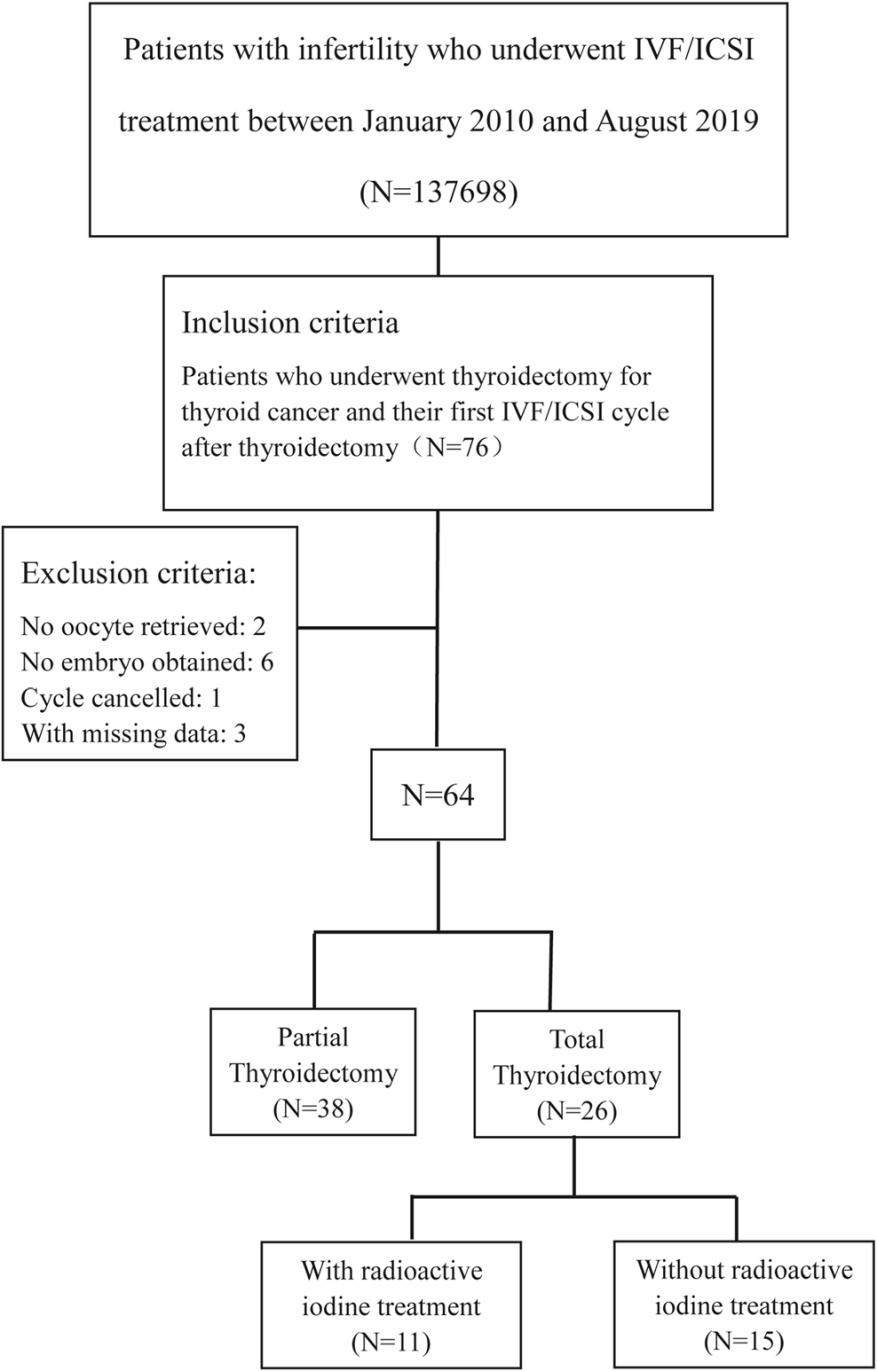
Flow chart of study cohort selection. IVF/ICSI, in vitro fertilization/intracytoplasmic sperm injection

A total of 137,698 women with infertility underwent IVF/ICSI treatment at the Reproductive Center of the Peking University Third Hospital between January 2010 and August 2019. Excluding patients >45 years old or complicated with other cancers, this study included 76 women with both infertility and a history of thyroidectomy for TC who underwent their first IVF/ICSI cycle after the surgery (Fig. 1). All the women had a histological diagnosis of papillary thyroid carcinoma. After excluding 2 patients with no oocyte retrieved, 6 patients with no embryo obtained, 1 patient canceling cycle, and 3 patients with missing data, 64 women with successful embryo transfer were included in our analysis, who were divided into two groups based on a history of partial and total thyroidectomy (*n*=38 and 26 women, respectively); 11 of the 26 women who underwent total thyroidectomy received RAIT.

### IVF treatment

All women included in the study underwent a standardized ovarian stimulation regimen and oocyte retrieval and fertilization followed by planned fresh or frozen embryo transfer. We treated 7, 12, 4, 33, and 8 women with ultra-long, long-term, short-term, antagonist, and mini-stimulation protocols, respectively. Recombinant human chorionic gonadotropin (HCG) was administered to trigger oocyte maturation when the mean diameter of at least two follicles reached 18 mm. Oocyte retrieval was performed 34 to 36 h after HCG administration. Insemination was performed 4 to 6 h after oocyte retrieval through either conventional IVF or ICSI, depending on the sperm quality. High-quality day 3 embryos or blastocysts were transferred to the women 3 or 5 days after oocyte retrieval. Some women underwent frozen embryo transfers because of a high risk of ovarian hyperstimulation syndrome, high progesterone levels, or personal reasons.

### Laboratory testing for thyroid function

Serum TSH, free thyroxine (FT4), thyroid peroxidase antibody (TPOAb), and thyroglobulin antibody (TGAb) levels were measured using a fully automated chemiluminescence immunoassay analyzer (ADVIA Centaur XP, Siemens Healthcare Diagnostics, Munich, Germany) before ovarian stimulation was performed. The TSH and FT4 reference values were 0.55–4.78 μIU/mL and 0.89–1.80 ng/dL, respectively. Based on the reference range provided by the manufacturer, TPOAb was defined as clinically positive at >60IU/mL, and TGAb was defined as clinically positive at >60IU/mL. In our study, patients positive for at least one of the thyroid antibodies were defined as thyroid antibody positivity. Of 64 patients in the TC group, 27 patients showed thyroid antibody positivity including 15 patients with co-positive for TPOAb and TGAb, 8 patients with isolated positive for TGAb, and 4 patients with isolated positive for TPOAb.

### Study outcomes

The IVF/ICSI outcomes in women who underwent the first IVF cycle and achieved embryo transfer were analyzed. Clinical pregnancy was defined as at least 1 gestational sac in the uterus identified on ultrasonography 35 days after embryo transfer. Live birth was defined as the delivery of at least one newborn that exhibits any sign of life, irrespective of gestation duration.

### Statistical analysis

Continuous and categorical variables are expressed as mean (standard deviation) and numbers (percentages), respectively. Student’s *t*-test and the chi-square test were used to compare differences in continuous and categorical variables, respectively, between the total and partial thyroidectomy groups. When continuous variables did not follow a Gaussian distribution, they were presented as median (interquartile range), and comparisons between groups were performed using the Mann–Whitney *U* test. To adjust for the relevant factors, multivariate logistic regression analysis was performed to calculate the odds ratios (ORs) with 95% confidence intervals (CIs). Results with a 2-tailed *P*-value <0.05 were considered significant. All statistical analyses were performed using SPSS version 24.0 (IBM corp., Armonk, NY).

## Results

The baseline characteristics of the women who underwent total and partial thyroidectomy are shown in Table 1. There were no differences in age, body mass index, duration and types of infertility, causes of infertility, basal hormone levels, antral follicle count, FT4 levels, and thyroid antibody positivity rates between the two groups. However, we observed a significant difference in TSH levels between the two groups. TSH levels were significantly lower in women who underwent total thyroidectomy than in women who underwent partial thyroidectomy (median [interquartile range]: 0.3 [0.1–0.9] vs. 0.7 [0.2–1.4], *P*=0.04). Table 2 compares the controlled ovarian stimulation (COS) protocols and IVF and embryo transfer data between women who underwent total and partial thyroidectomy. There were no differences in the COS protocols, gonadotropin dose, number of COS days, HCG trigger day hormone levels, and number of good-quality embryos between the two groups. The number of retrieved oocytes was significantly lower in women who underwent total thyroidectomy than in women who underwent partial thyroidectomy (median [interquartile range]: 7.0 [6.0–11.3] vs. 10.5 [6.0–17.3], *P*=0.047).

**Table 1:** Baseline characteristics between women with total thyroidectomy and partial thyroidectomy

Characteristics	Total thyroidectomy (*N*=26)	Partial thyroidectomy (*N*=38)	*P*-value
Age, mean (SD), y	34.9 (3.3)	33.0 (4.8)	0.09
Body mass index, mean (SD), kg/m^2^	23.3 (3.3)	23.0 (2.8)	0.68
Duration of infertility, median (IQR), y	3.0 (1.4–5.3)	4.0 (2–5.3)	0.24
Type of infertility, no. (%)			
Primary	13 (50.0)	22 (57.9)	0.53
Secondary	13 (50.0)	16 (42.1)	
Causes of infertility, no. (%)			
Male factors	12 (46.2)	16 (42.1)	0.75
Female factors			
Tubal factor	11 (42.3)	12 (31.6)	0.38
Polycystic ovary syndrome	4 (15.4)	9 (23.7)	0.42
Endometriosis	1 (3.8)	3 (7.9)	0.64^#^
Unknown factors	3 (11.5)	5 (13.2)	>0.99^#^
Basal FSH, median (IQR), mIU/mL	7.2 (5.6–8.4)	7.1 (4.9–9.2)	0.54
Basal LH, median (IQR), mIU/mL	3.3 (2.4–4.3)	3.2 (2.5–4.2)	0.89
Basal estradiol, median (IQR), pmol/L	163.5 (141.8–199.5)	163 (119.5–232.5)	0.92
Antral follicle count in both ovaries, mean (SD)	10.5 (5.8–13.3)	12.0 (8.8–16.3)	0.09
FT4, median (IQR), ng/dL	1.4 (1.3–1.7)	1.5 (1.3–1.7)	0.99
TSH, median (IQR), mIU/L	0.3 (0.1–0.9)	0.7 (0.2–1.4)	0.04*
No. of thyroid antibody positivity^a^, no. (%)	8 (30.8)	19 (52.8)	0.09

*SD* standard deviation, *IQR* interquartile range, *FSH* follicle-stimulating hormone, *LH* luteinizing hormone, *FT4* free thyroxine, *TSH* thyroid-stimulating hormone

*P<0.05

^#^Fisher’s exact test

^a^Patients positive at least one of the thyroid antibodies were defined as thyroid antibody positivity. TPOAb was defined as clinically positive at >60IU/mL, and TGAb was defined as clinically positive at >60IU/mL

**Table 2:** Protocols of controlled ovarian stimulation and data of in vitro fertilization and embryo transfer between women with total thyroidectomy and partial thyroidectomy

Characteristics	Total thyroidectomy (*N*=26)	Partial thyroidectomy (*N*=38)	*P*-value
Protocols of controlled ovarian stimulation, no. (%)			
Ultralong GnRH agonist	2 (7.7)	5 (13.2)	0.88
Long GnRH agonist	4 (15.4)	8 (21.1)	
Short GnRH agonist	2 (7.7)	2 (5.3)	
GnRH antagonist	15 (57.7)	18 (47.4)	
Mini stimulation	3 (11.5)	5 (13.2)	
Gonadotropin dose, median (IQR), IU	2400.0 (1800.0–2925.0)	2062.5 (1350.0–3318.8)	0.39
No. of days of ovarian stimulation, median (IQR)	10 (9–11)	10 (9–12)	0.38
LH on HCG trigger day, median (IQR), mIU/mL	1.7 (1.1–3.3)	1.3 (0.5–2.9)	0.20
Estradiol on HCG trigger day, median (IQR), mIU/mL	5016.5 (3172.5–8436.5)	5914.0 (3983.8–14217.8)	0.22
Progesterone on HCG trigger day, median (IQR), pmol/L	2.1 (1.5–3.0)	2.0 (1.5–3.2)	0.92
No. of retrieved oocytes per cycle, median (IQR)	7.0 (6.0–11.3)	10.5 (6.0–17.3)	0.047*
Fertilization, no. (%)			
IVF	15 (57.7)	21 (55.3)	0.85
ICSI	11 (42.3)	17 (44.7)	
No. of good-quality embryos per cycle, median (IQR) ^a^	3.0 (1.0–4.0)	3.0 (2.0–6.3)	0.38
No. of embryos transferred, no. (%)			
1	8 (30.8)	7 (18.4)	0.33
2	18 (69.2)	30 (78.9)	
3	0	1 (2.6)	

*GnRH* gonadotropin-releasing hormone, *IQR* interquartile range, *LH* luteinizing hormone, *HCG* human chorionic gonadotropin, *IVF* in vitro fertilization, *ICSI* intracytoplasmic sperm injection

*P<0.05

^a^The embryos were evaluated on the third day after fertilization. Good-quality embryos were developed from two pronuclei zygotes and met the following criteria: (1) had more than five blastomeres; (2) size difference was less than 20%; and (3) fragmentation was less than 50%

A total of 22 women achieved clinical pregnancy, with no significant differences between the total and partial thyroidectomy groups (23.1% vs. 44.7%, *P*=0.076; Table 3). One patient in the partial thyroidectomy group had an early pregnancy abortion. No significant differences were observed in the live birth rates (23.1% vs. 42.1%, *P*=0.115; Table 3) between the total and partial thyroidectomy groups. However, after adjusting for age, TSH levels, RAIT, thyroid antibody positivity and protocols of COS, women who underwent partial thyroidectomy had 7- and 6-fold higher clinical pregnancy and live birth rates, respectively, than those who underwent total thyroidectomy (OR, 7.70 and 6.40; 95% CI, 1.22–48.77 and 1.02–40.34; *P*=0.030 and 0.048, respectively; Table 5).

**Table 3:** Pregnancy outcomes between women with total thyroidectomy and those with partial thyroidectomy

Outcomes	Total thyroidectomy (*N*=26)	Partial thyroidectomy (*N*=38)	*P*-value
Clinical pregnancy^a^, no. (%)	6/26 (23.1)	17/38 (44.7)	0.076
Live birth^b^, no. (%)	6/26 (23.1)	16/38 (42.1)	0.115

^a^Clinical pregnancy was defined as at least one gestational sac in the uterus at 35 days after embryo transfer as identified on ultrasonography

^b^Live birth was defined as delivery of at least one living fetus beyond 28 weeks of gestation

No significant difference was observed in clinical pregnancy and live birth rates between the RAIT and non-RAIT groups (27.3% vs. 37.7% and 27.3% vs. 35.8%, *P*=0.732 and 0.735, respectively; Table 4) even after adjusting for age, TSH levels, surgical treatment, thyroid antibody positivity and protocols of COS (OR, 1.66 and 1.96; 95% CI, 0.18–15.61 and 0.20–18.91; *P*=0.660 and 0.559, respectively; Table 5).

**Table 4:** Pregnancy outcomes between women in the RAIT and non-RAIT groups

Outcomes	RAIT (*N*=11)	Non-RAIT (*N*=53)	*P*-value
Clinical pregnancy^a^, no. (%)	3/11 (27.3)	20/53 (37.7)	0.732
Live birth^b^, no. (%)	3/11 (27.3)	19/53 (35.8)	0.735

*RAIT* radioactive iodine treatment

^a^Clinical pregnancy was defined as at least one gestational sac in the uterus at 35 days after embryo transfer as identified on ultrasonography

^b^Live birth was defined as delivery of at least one living fetus beyond 28 weeks of gestation

**Table 5:** Multivariate logistic regression analysis of factors associated with pregnancy outcomes in women with TC

Factors	Clinical pregnancy		Live birth	
	OR (95% CI)	*P*-value	OR (95% CI)	*P*-value
Age (years)				
<35 (reference)	NA	NA	NA	NA
>35	0.41 (0.09–1.97)	0.268	0.28 (0.06–1.42)	0.125
TSH (μIU/ml)				
<0.5 (reference)	NA	NA	NA	NA
>0.5	0.28 (0.06–1.25)	0.096	0.32 (0.07–1.41)	0.133
Surgical options				
Total thyroidectomy	NA	NA	NA	NA
Partial thyroidectomy	7.70 (1.22–48.77)	0.030^*^	6.40 (1.02–40.34)	0.048^*^
Radioactive iodine treatment	1.66 (0.18–15.61)	0.660	1.96 (0.20–18.91)	0.559
Thyroid antibody positivity^a^	0.22 (0.05–0.95)	0.042^*^	0.27 (0.06–1.12)	0.071
Protocols of COS				
Mini stimulation (reference)	NA	NA	NA	NA
Ultralong GnRH agonist	-	0.999	-	0.999
Long GnRH agonist	1.51 (0.21–11.00)	0.683	2.97 (0.36–24.21)	0.310
Short GnRH agonist	1.96 (0.14–27.67)	0.619	3.40 (0.22–51.90)	0.379
GnRH antagonist	1.09 (0.19–6.37)	0.926	2.05 (0.31–13.54)	0.455

*TC* thyroid cancer, *OR* odds ratio, *TSH* thyroid-stimulating hormone, *CI* confidence interval, *COS* controlled ovarian stimulation

*P<0.05

^a^Patients positive at least one of the thyroid antibodies were defined as thyroid antibody positivity. TPOAb was defined as clinically positive at >60IU/mL, and TGAb was defined as clinically positive at >60IU/mL

Multivariate logistic regression analysis was performed to evaluate several factors associated with the IVF/ICSI outcomes in women who received TC treatment. The age and TSH levels showed no association with the clinical pregnancy and live birth rates (Table 5). Thyroid antibody positivity was an important risk factor for IVF/ICSI outcomes. Compared with women without thyroid antibody positivity, those with thyroid antibody positivity had significantly lower clinical pregnancy (OR, 0.22; 95% CI, 0.05–0.95; *P*=0.042).

## Discussion

To the best of our knowledge, our study is the first to demonstrate the differences in IVF/ICSI outcomes between women who underwent total and partial thyroidectomy. After adjusting for age, TSH levels, thyroid antibody positivity, RAIT and protocols of COS, we found that clinical pregnancy and live birth rates were significantly lower in women who underwent total thyroidectomy than in those who underwent partial thyroidectomy. The mechanism triggering this difference remains unknown; however, hypothalamus-pituitary-thyroid (H-P-T) axis deficiency and hypocalcemia may be involved. Sufficient thyroid hormone availability is an essential regulator in fetal growth and development. Fetal demand for thyroid hormone during early pregnancy solely depends on the supply transferred from maternal thyroid hormone because the fetal thyroid gland is non-functional until the 18th week of pregnancy. Thus, the demand for thyroid hormone availability is significantly increased during early pregnancy. Human chorionic gonadotropin (HCG), produced specifically and rapidly in early pregnancy, stimulates the thyroid gland via binding to the TSH receptor, thereby triggering an elevation in serum FT4 levels and a subsequent feedback suppression in TSH concentration. The HCG-mediated thyroid response is an important way safeguarding the adequate thyroid hormone [16, 17]. Total ablation of thyroid gland in patients with total thyroidectomy leads to the damage in the H-P-T axis and the deficiency in the secretion of thyroid hormone and the feedback suppression of TSH, which may induce an imbalance in thyroid hormone availability. Aside from the H-P-T axis damage, hypocalcemia and vitamin D deficiency may also be associated with adverse IVF/ICSI outcomes. Filippo et al. reported a nearly 3-fold higher hypocalcemia risk in patients who underwent total thyroidectomy than in patients who underwent lobectomy [18]. Vitamin D plays a pivotal role in regulating calcium metabolism, and a recent study showed a significant association between postoperative hypocalcemia and low preoperative vitamin D levels in patients after total thyroidectomy [19]. Many studies have reported on the correlation between vitamin D deficiency and decreased clinical pregnancy and live birth rates after IVF/ICSI [20, 21]. Lower calcium and vitamin D levels in women with total thyroidectomy may be an important risk factor associated with IVF/ICSI outcomes, and appropriate calcium and vitamin D supplement doses may be considered before commencing IVF/ICSI treatment in women with TC.

RAIT is widely used for postsurgical TC treatment. Recently, an increasing number of studies have focused on the effect of RAIT on reproductive function and fertility in women of childbearing age. Ceccarelli et al. suggested that RAIT might contribute to follicular atresia and menopause induction [14]. A recent meta-analysis that included 22 studies demonstrated that RAIT negatively influenced the menstrual cycle in the first year after treatment but was not associated with diminished long-term fertility [22]. Several studies have reported a correlation between RAIT and pregnancy outcomes; however, the results are controversial. Wu et al. and Anderson et al. reported that RAIT did not affect the birth rate, while another cohort study found that RAIT was significantly associated with a lower successful delivery rate [11–13]. Recently, Kim et al. conducted a large-scale, nationwide cohort study that evaluated whether the interval between conception and RAIT was associated with an increased risk of adverse pregnancy outcomes. The study found that conception that occurred less than 6 months after RAIT was associated with higher congenital malformation and miscarriage risks; however, the association was not observed in the 6–11 month interval group [23]. There are no studies that have reported an appropriate interval between RAIT and IVF/ICSI treatment. In our study, all women underwent IVF/ICSI treatment more than 6 months after RAIT, and we identified no significant correlation between RAIT and lower clinical pregnancy and live birth rates. Thus, based on our findings, IVF/ICSI treatment may be ideal for women who have previously undergone RAIT when IVF/ICSI treatment is performed more than 6 months after RAIT.

Our multivariate logistic regression analysis showed that the presence of thyroid antibody positivity was significantly associated with decreased clinical pregnancy rates in women with TC treatment. Some studies have reported that patients with thyroid antibody positivity were more likely to develop TC. For example, Qin et al. reported a higher prevalence of elevated TGAb and TPOAb in patients with TC than in those with benign thyroid nodules [8]. Another prospective study recruited 2100 patients with thyroid nodules and reported a significantly increased OR of raised TGAb levels in the presence of TC, further conferring TGAb with a role in predicting the TC risk [24]. Kratky et al. and Wong et al. reported a significant association between thyroid antibodies including TPOAb and TGAb and the risk of TC based on the patients after fine needle aspiration biopsy of thyroid nodule and identified thyroid antibody positivity as a risk factor of malignancy [9, 10]. Although no studies have evaluated the impact of thyroid antibodies on IVF/ICSI outcomes in infertile women with TC, several studies have demonstrated an association between the presence of thyroid antibodies and IVF/ICSI outcomes in women with infertility. Karacan et al. analyzed the ICSI outcomes in 253 women undergoing ICSI-embryo transfer cycles and found that the presence of thyroid antibodies did not affect the ICSI outcome in euthyroid and antiphospholipid antibody-negative women [25]. In a meta-analysis that included 12 studies, Busnelli et al. provided a comprehensive analysis of the association between thyroid antibody positivity and IVF/ICSI outcomes [7]. They concluded that the presence of thyroid antibodies had a detrimental effect on pregnancy outcomes, with an increased miscarriage risk and a decreased live birth frequency. Our study is the first to provide evidence that thyroid antibody positivity is an independent risk factor for adverse IVF/ICSI outcomes in women with TC treatment. However, the mechanism through which thyroid antibodies affect pregnancy outcomes remains unclear. Our previous study demonstrated that treatment with levothyroxine did not reduce the miscarriage rates or increase the clinical pregnancy and live birth rates of women undergoing IVF-ET who had normal thyroid function and tested positive for thyroid autoantibodies [26]. Dysfunctions in immunological regulation may be involved in the mechanism and should be explored further [27].

This study had several strengths. To date, this study investigates the largest number of infertile patients with TC who underwent IVF/ICSI treatment. Second, to the best of our knowledge, this is the first study to evaluate the association between TC treatment and IVF/ICSI outcomes. The major limitation is the retrospective design of this study, which may cause information bias. The thyroid hormone and TSH levels during the period of pregnancy were not obtained. Further prospective studies with a larger sample size are required to confirm these findings. In conclusion, this study evaluated the effects of different types of surgical procedures and RAIT on IVF/ICSI outcomes and analyzed the possible risk factors associated with adverse IVF/ICSI outcomes in women with TC treatment. Our study showed that clinical pregnancy and live birth rates were lower in women who underwent total thyroidectomy than in women who underwent partial thyroidectomy. Our study suggests that clinicians should carefully consider an optimal surgical procedure for women of childbearing age with TC. However, we found that RAIT did not affect IVF/ICSI outcomes if the IVF/ICSI treatment occurred more than 6 months after RAIT. Furthermore, our study showed that thyroid antibody positivity was an independent risk factor for adverse IVF/ICSI outcomes.

## Data Availability

The datasets used or analyzed during the current study are available from the corresponding author on reasonable request.
